# Affective Scaffoldings as Habits: A Pragmatist Approach

**DOI:** 10.3389/fpsyg.2021.629046

**Published:** 2021-03-25

**Authors:** Laura Candiotto, Roberta Dreon

**Affiliations:** ^1^Department of Philosophy and Humanities, Institute of Philosophy, Free University of Berlin, Berlin, Germany; ^2^Department of Philosophy and Cultural Heritage, Ca' Foscari University of Venice, Venice, Italy

**Keywords:** affective habits, affective scaffoldings, situated affectivity, John Dewey, Pragmatism, sensibility, emotion, habit crisis

## Abstract

In this paper, we provide a pragmatist conceptualization of affective habits as relatively flexible ways of channeling affectivity. Our proposal, grounded in a conception of sensibility and habits derived from John Dewey, suggests understanding affective scaffoldings in a novel and broader sense by re-orienting the debate from objects to interactions. We claim that habits play a positive role in supporting and orienting human sensibility, allowing us to avoid any residue of dualism between internalist and externalist conceptions of affectivity. We provide pragmatist tools for understanding the environment's role in shaping our feelings, emotions, moods, and affective behaviors. However, we contend that in addition to environment, the continuous and recursive affective transaction between agent and environment (both natural and cultural) are also crucially involved. We claim that habits are transformative, which is especially evident when we consider that emotions are often the result of a crisis in habitual behavior and successively play a role in prompting changes of habits. The final upshot is a conceptualization of affective habits as pervasive tools for feelings that scaffold human conduct as well as key features in the transformation of behaviors.

## Introduction

Habitual ways of doing, feeling, thinking, and interacting are pervasive in human behaviors.

In this paper, we will provide a pragmatist conceptualization of human habits as relatively stable, i.e., more or less flexible, ways of channeling resources that come from both the environment and the organism [Dreon, Human Landscapes: Contributions to a Pragmatist Anthropology, under review, ch. 4]. The Classical Pragmatists, in contrast to the behaviorist account of habits as a mechanical reaction to stimuli, stressed the creative power of habits to scaffold human behaviors. According to this view, habits play a positive role in supporting and orienting human sensibility, as well as in sustaining and nourishing cognition. They are the skeleton of human social behaviors.

We will frame our approach within the contemporary debate on affective scaffoldings and specifically look at “affective habits” as relatively stable, i.e., more or less flexible ways of channeling affectivity. We will thereby provide pragmatist tools for understanding the environment's role in orchestrating our feelings, emotions, moods, and affective behaviors. We will contend that in addition to the environment's influence on the agents' behaviors, the continuous and recursive affective transaction between agent and environment (both natural and cultural) also plays a crucial role. This constitutive interdependence between agent and environment is what makes the very same affective gestures habitual. It is also what makes habits essentially social. They are not mere properties of individuals but also modes of group behavior, such as the enthusiastic support football fans show when their team is in trouble or the pernicious stereotyping of minorities.

In a nutshell, we suggest considering affectivity as both scaffolded by habits and scaffolding habits. On the one hand, we claim that affectivity is a permanent feature of the active human experience of the world, supported by habits. We conceptualize habits as relatively stable—sometimes more flexible, sometimes more routine—ways of selecting stimuli, identifying salient features, moving toward an object of interest, and distance oneself form or getting closer to someone else. We then shed light on the notion of “affective habits.” In doing so, we are not claiming in favor of a new entity, beyond “emotions,” “moods,” and all the other items on the list of the affective states. Rather, we take “affective habits” as a hermeneutical tool that enables us to emphasize that affectivity is propulsive in involving and favoring relatively regular transactions between embodied agents and their natural as well as culturally shared environment. On the other hand, we claim that affectivity gives rise and nourishes more or less standardized practices, consolidated ways of facing circumstances, socially shaped ways of praising and blaming that remain largely unreflective. In this way, affectivity is not just scaffolded by habits but it also scaffolds habits.

This is evident in the breaking of previous habits of acting, feeling, and valuating. Although habits, including affective ones, mostly work unreflectively, their breaking elicits a more reflective analysis of the issue at stake and the active search for a new more intelligent accommodation, namely a new form of attunement with changed circumstances. Furthermore, although habits—particularly habits of feeling—are often pre-personally and not consciously acquired, their crisis pushes agents to become aware of what they are doing, to become conscious of their own dispositions toward others and of their responsibility within a previously unproblematic transaction. Consequently, habit change and habit crisis show a crucial transformative power.

In order to explain all of this in detail, some preliminary steps are required—as well some patience on the reader's part. First of all, we frame our proposal within the contemporary research on affective scaffoldings (section On Affective Scaffoldings and Their Theoretical Background). After developing the pioneering investigation on affective scaffoldings carried out by Colombetti and Krueger (2015), we will contend that affective scaffoldings are habits. This will allow us to understand affective scaffoldings in an innovative and broader sense by re-orienting the debate from objects to interactions. The notion of habits makes it possible to overcome certain limitations of the contemporary literature on affective scaffoldings, such as the predominant focus on material culture and the function of emotion regulation. We will then list our pragmatist correctives, which is to say that we will present some reasons in support of our choice to speak about sensibility instead of affectivity and to place emotions within the continuum of sensibility itself give some preliminary explications about affectivity, sensibility, and emotions (section Affectivity, Sensibility, and the Emotions). In section A Deweyan Conception of Habits we will provide a fresh introduction to the pragmatist view of habits with a focus on the work of John Dewey. Our pragmatist correctives will then be analytically described in the subsequent sections. In section Affective habits, we deal with the role of habits in scaffolding affective behaviors. In section Habit Crisis and Emotions, we clarify why habits can be transformative via an explication of emotions as both a result of a crisis in habitual behavior and an active agent in prompting later habit change. The main features and functions of affective habits that we brought into the spotlight will be recapitulated in the Conclusion. The final upshot is a conceptualization of affective habits as pervasive tools for feelings that scaffold human conduct as well as key features in the transformation of behaviors.

## On Affective Scaffoldings and Their Theoretical Background

With their ground-breaking and programmatic paper “Emotions in the Wild,” Griffiths and Scarantino (2008) have designed a new naturalistic model for understanding emotions as both social and intersubjective phenomena. This model is considered the best example of what is now called “situated affectivity,” according to which emotions are strategic moves within environmental structures, i.e., active engagements with the social world. Griffith and Scarantino avoid any internalist conceptualisations of emotion, which view emotions as environmentally independent internal states or processes that marginalize the environment's role (Griffiths and Scarantino, 2008, p. 437)^1^. As a consequence, a situated approach to affectivity is thus one that recovers the crucial role of the environment in producing, shaping, and managing emotions. This means that the environment both influences and is influenced by the unfolding of emotions (Griffith and Scarantino, p. 438). Griffith and Scarantino have especially stressed the social embeddedness of emotions, arguing that they are social signals that constantly reframe relationships. The conceptual framework is thus efficiently bi-directional and has at its core the notion of strategic interaction.

As we will see in a moment, Dewey suggested an analogous shift for avoiding internalist conceptions of habits nearly a century ago, framing his theory of habits as an introduction to social psychology (Dewey, 1983). Along similar lines, at the beginning of the twentieth century, Georg Herbert Mead strongly favored the development of social psychology as a necessary “counterpart to physiological psychology” (Mead, 2011, p. 9).

Acknowledging that the human environment is natural as well as social and cultural, Griffith and Scarantino have offered an efficient model for understanding the relationship between evolution and social context in producing an emotion. Drawing from the extended mind hypothesis (Clark and Chalmers, 1998), they have stressed the active role of an “environmental scaffolding” in supporting emotions. Of particular significance to them is the social embeddedness of emotions, specifically as social signals that are constantly reframing relationships and are therefore both synchronic (supporting an emotional episode) and diachronic (supporting the development of a repertoire of emotional abilities) strategic moves. The active role played by the environment should not be simply construed as a causal trigger of affective experience. Rather, the environment offers action-possibilities in the form of emotions. Drawing from Scarantino's previous work on affordances (Scarantino, 2003), Griffith and Scarantino have thus offered an action-oriented view of non-conceptual emotional content. In emotion, the environment is represented in terms of what it affords^2^.

Drawing upon the notion of “environmental scaffolding,” Colombetti and Krueger (2015) have defined “affective scaffoldings” as those resources that set up, drive, and regularly contribute to affective regulation^3^. Emotion-regulation is a fundamental process that humans undertake to shape and manage their mental lives. The externalist approach to emotion-regulation claims that external resources are employed to adjust and manage human feelings—as in the case of going for a good run when one is angry and trying to appease an intense feeling of hostility. The core idea is that specific resources can balance human affective life if they are integrated into structured and repeated practices of interaction. Krueger (2018) has further distinguished three types of affective scaffoldings. There are embodied affective scaffoldings (1), in which the affective experience is regulated by a range of physical processes distributed throughout our bodies, not only in the brain but also in, for example, the digestive or hormonal systems. Just think of how sleepy and calm you feel after having eaten a tasty plate of spaghetti, or the intermittent phases of sorrow and anxiety that some women feel at different phases of their menstrual cycle. We then have social affective scaffoldings (2), in which socially distributed feedback loops regulate the affective dynamics of individuals and groups. This is the case, for example, with family dinners, parties and celebrations that boost affective bonds, or with a range of therapeutic settings where intersubjective activities regulate affectivity. The last case is that of material affective scaffoldings (3), in which affectivity is regulated by the material culture that is made up of particular objects and environments, as in the case of a wedding ring which reinforces one's trust in and fidelity toward one's partner, or of a skillfully designed house with east-facing windows that allow for ample morning sunlight, which ensures that you get out of bed with more energy.

Despite Krueger's postulation that there are three types of affective scaffolding, most philosophical research on environmental scaffoldings has been devoted to the last case of material culture (Carter et al., 2016; Krueger and Colombetti, 2018; Piredda, 2019; Colombetti, 2020). There are exceptions, of course, but a tendency to see affective scaffoldings as material things that help humans regulate affectivity is quite common and widespread^4^. The function of affective scaffoldings has consequently been identified as mostly affective regulation. Although this function is undoubtedly crucial, we think other functions should also be taken into account. As an example, let us take the case of an academic browsing a bookshelf as a reward after a hard day's work, thereby enhancing their sense of self (Piredda, 2019; see also Dings, 2017: 691). This function may be understood as a specific type of affective regulation—in this case, boosting self-esteem and self-efficacy. It could be objected that this does not contribute anything new to the debate. While we grant that self-enhancement could be a case of affective regulation, we still think that Piredda is pointing in the right direction. Instead of listing functions performed by affective scaffoldings, we should shift the focus from (material) objects to actions that are occurring, i.e., the kinds of transactions—mostly habitual ones—through which affectivity is scaffolded. We can thereby enlarge and partly redirect the perspective to appreciate the pervasive presence of “affective scaffoldings” in our affective life. This means looking at affective habits as a broader notion that can explain how our affectivity works in its entanglement with an environment.

“Affective scaffoldings” is a unique hermeneutical tool that plays a fundamental role in drawing attention to the continuous and recursive interactions with the environment shaping affectivity. But this notion implies reducing the whole matter to the use of a resource, be it a material item, a person, or a culture. We would like to avoid the risk of this implication and will therefore look at affective habits in terms of transactions and the way they unfold, which involves constitutive relationships between different aspects of the same environment, including organic features and physical constraints as well as social processes and cultural meanings. We claim that living beings' transactions within their environment occur affectively and mostly habitually, or in conjunction with a habit crisis. We suggest this conceptual shift not only out of phenomenological accuracy but also because it seems to us that affective scaffoldings reproduce a dualistic residue of the consumerist user-resources relationship that overshadows their social form^5^. We wish to argue that looking at affective scaffoldings as habits can help avoid the latter^6^.

For us, the important claim in Dewey's pragmatist perspective is that human habits are intelligent behaviors rooted at the point of intersection between socio-cultural features and biological resources. Human habits are about transactions of living organisms that are embedded in an environment that is natural, inherently social, and culturally shared since birth. We argue that this is why habits are more than skillful actions or unreflective repetitions of a previously conscious voluntary act and are pervasive in human behaviors. Habits are behaviors that result from the transactions between humans and environments over their history. Affective habits in particular scaffold our feelings, perceptions, interactions, and even decision-making processes. Looking at habits thus requires a radical approach: we must hold fast to the basic assumptions of situated affectivity and extend them to a general theory of making human affectivity. It may be objected that in doing so, we are abandoning a clear-cut notion such as “affective scaffolding” in favor of a less specific one, namely the notion of “habits,” and that as a consequence, we will be left with nothing but approximations or, even worse, generalizations. We are confident this is not the case, and the main task of this paper is precisely to show how and why this is so.

Another objection might arise from the fact that habits are already mentioned in the literature on affective scaffoldings (Colombetti, 2016; Maiese, 2016; Slaby, 2016; Krueger and Colombetti, 2018). Thus, it could be objected that our proposal is not really innovative and that habits have already been conceptualized within the framework of affective scaffoldings. However, we are not just making a notion that is already prevalent in this field of study more explicit and prominent. We also seek to correct a mistaken view of habits as a conservative social power. In the philosophical literature about affective scaffoldings, affective habits are unfortunately mostly understood as simple ways of passively doing things out of custom or routine (see, for example, Colombetti and Krueger, 2018, p. 224). As we will see in the next sections, however, emotions can be a powerful source of change, and habits can thus be reshaped and altered. Affective habits are not merely customary emotional responses; they do not simply allow the agent to become absorbed by an affective niche, as is sometimes the case with mind invasion phenomena (Slaby, 2016). In special situations (ones in which a crisis triggers a revision of habits: see section Habit Crisis and Emotions), habits are open to being amended.

The last critical point we find in these studies is that affective habit incorporation is detected especially in the body (Colombetti, 2016). This effort is certainly praiseworthy, and we think that affective gesture can be interpreted as affective habits as well^7^. But our conceptualization of affective habits will lead to a broader horizon of habitual actions, one in which affective habits are also habits of perceiving, interpreting, and thinking, as we will clarify in section Affective Habits^8^. Through John Dewey's insightful criticism of the mind-body dualism (section A Deweyan Conception of Habits), we will be able to appreciate both the embodied and cognitive dimension of affective habits in the use of organic and environmental (natural and social) resources for scaffolding human affectivity.

To sum up, we need to address the following question: Why should we look at affective scaffoldings as habits in pragmatist terms? First, we should do so to avoid the risk of reducing the relevance of affective scaffoldings to the use of environmental resources to carve out human behaviors. This has led researchers to focus on the use of objects, which risks promoting a consumerist user-resources relationship, if only unwittingly. Second, we should adopt a pragmatist perspective in order to emancipate ourselves from a passive and routine view of scaffolded affectivity so as to bring the habits' power of transformation into the spotlight. Finally, we should take this approach in order to better appreciate affective habits' cognitive function, and to avoid reducing them to a bodily matter.

## Affectivity, Sensibility, and the Emotions

In this approach of conceptualizing human affectivity as something pervasively scaffolded by habits, we owe our readers some preliminary explanations concerning the notions of affectivity, sensibility, and the emotions that ground our interpretive claim. In the previous section, we set the scene by framing our discussion within the literature on affective scaffoldings. Here, we will make our conceptual tools explicit.

Affectivity is a broader phenomenon than just emotions^9^. It generally refers to “a *lack of indifference*, and rather a *sensibility* or *interest* for one's existence” (Colombetti, 2014, p. 1); it is “the capacity, as well as the conditions of being affected (literally, “done something”) by something,” specifically never being “deprived of any interest, concern, or care for one's existence and/or world” (Colombetti, 2014, p. 5). Affectivity “permeates the mind, necessarily and not contingently” (Colombetti, 2014, p. 1). This conception has at least two significant consequences. On the one hand, affectivity cannot be considered the mere association of discrete parts; it is rather a constant feature of human-environment interactions^10^. From this point of view, emotions must be understood against the background of affectivity and not the other way round. On the other hand, we can no longer assume a primarily non-affective and purely cognitive mind which records merely descriptive states of things and eventually colors them through subjective feelings (Nussbaum, 2001).

Pragmatism helps in a different way by developing the idea of the pervasive nature of affectivity. “The isolation of traits characteristic of objects known, and then defined as the sole ultimate realities, accounts for the denial to nature of the characters which make things lovable and contemptible, beautiful and ugly, adorable and awful” (Dewey, 1988a, p. 28). This means that agents do not first apprehend objects as neutral and only subsequently assess them as lovable or contemptible. On the contrary, objects are perceived as lovable and contemptible. An item's quality of being lovable emerges in the interaction with a subject who experiences it as lovable. But this is not the one-sided, powerful creation of reality by the subject, as per Idealism. Drawing upon James's radically empiricist claim that relations must be assumed to be as real as discrete entities (James, 1976), Dewey stressed that being lovable is a feature of the real relation existing between the baby and its blanket; it is not a secondary property of the blanket because it is inscribed in living beings' dependence on a context by which they are always affected in one way or another^11^. When needed—for example, when the blanket is no longer at hand—we reflectively return to our primarily holistic experience and discriminate between the physical features of a situation in order to find a way out of a crisis—in this example, to recover the lost blanket. But perception is already affectively laden, which is to say that it is charged with a meaning concerning the life of the organism insofar as it is constituted through the interactions it has with the environment to which it belongs and on which it depends. In a nutshell, the focus is on the interaction between the agent and the environment from which the object's features and the agent's feelings emerge as something “in-between” (Candiotto, 2019).

In light of this, we have chosen to use the word “sensibility,” because this term traditionally has two different meanings and/or usages in ordinary language and technical debates. “Sensibility” refers both to experience as perception through the senses and to experience involving feelings, emotions, affective valuations, and sensually-driven interests and dispositions (see Marcuse, 1955). One of us has even suggested assuming that this ambivalence of the word is a positive feature rather than a linguistic flaw derived from the vagueness of ordinary speech (Dreon, Human Landscapes: Contributions to a Pragmatist Anthropology, under review). With respect to “affectivity,” “sensibility” allows us to explicitly regard organic-environmental transactions as transactions that structurally intertwine sensory-perceptual and affective aspects, as opposed to envisaging these as two independent dimensions that only interact at a subsequent stage^12^. We adopt a definition of sensibility that shares John Dewey's approach to experience, which primarily refers to the dependence of organic life on transactions with its environment. Therefore, we define sensibility as “selective exposure to the environment and an active capacity of feeling to discriminate between favorable and negative aspects by organisms whose primary experience of the surrounding environment is socio-cultural. This is due to the organic conditions of emphasized interdependence from others characterizing the human form of life” (Dreon, Human Landscapes: Contributions to a Pragmatist Anthropology, under review).

In *Knowing and the Known*, Dewey, with Arthur Bentley, stated that the word “transaction” could clarify “the full organic-environmental situation” with reference to the knowing process (Dewey and Bentley, 1964). In other words, we should not regard the organism and the environment as two distinct entities that only interact at a subsequent stage, but rather as two mutually determining processes. Organic-environmental transactions should be considered as both epistemologically and ontologically primary given that, an organism is both thoroughly constituted by environmental resources and a part of the environment, itself entering the dynamic by shaping the environment and thus transforming it from within, albeit to a greater or lesser degree^13^. Consequently, sensibility appears to be grounded in the very conditions of organic life and dependent on an environment for its own survival and flourishing.

Against this background, emotions can and should be considered to be “emotional episodes” (Colombetti, 2014, p. 25) within the continuity of affectively charged organic–environmental transactions. Basically, we wish to adopt a pragmatist view of emotions (Dreon, 2019). Emotions are not external expressions of an alleged interior state of mind, but emotive gestures of the human (inter)actions that are embedded in and responding to a specific environmental context characterized by a sharpened level of sociality. Already at the end of the 19th century, James criticized the idea of an alleged “mind stuff” which pre-exists its alleged bodily expressions (James, 1884). Largely foreshadowing the situated view of emotions we encountered in the previous section, Dewey emphasized that emotions are always embedded in an environment, to which they always make a variety of “prepositional references” (Dewey, 1971): We are afraid *of* something, happy *because of* a specific event or relationship, annoyed or charmed *by* someone (Dewey, 1981). In particular, emotions are modes of inter-personal attunement, and function as tools for coordinating social conduct (Mead, 2011). They also provide the organism with a kind of affective “proto-valuation”^14^ of environmental conditions connected to the (weaker or stronger) impact that a natural and/or social feature of the environment can have on the organism's life—a kind of qualitative appraisal that can later become an object of reflection and judgment^15^.

In what follows, we investigate the varieties of ways through which sensibility and emotions are intimately connected with habits, given an ecological conception of habit inspired by Dewey's statement that they are functions of the organism as well as the environment (Dewey, 1983). By “ecological,” we mean that both organic and environmental features are equally essential traits of the constitution of habits. We should not assume that merely external constraints condition basically internally determined behaviors. This point is grounded in a Deweyan view that the human organism is part of the environment and that it acts in it and through it. Consequently, distinctions between a living being and the environment it belongs to should be regarded as being operative and context-dependent, and not an ontological divide (Dewey, 1989, p. 19, see Alexander, 1987, p. 135 on this point).

## A Deweyan Conception of Habits

In order to support our claim that sensibility and the emotions are scaffolded through habits, we will now outline a pragmatist conception of habit derived from Dewey^16^. In recent years, enactivist scholars have devoted specific attention to habits as a key concept for understanding human behavior and agency (see Ramírez-Vizcaya and Froese, 2019 for an overview), providing an opportunity to overcome the standard behavioristic account of habit as an automatization derived from the mere repetition of a stimulus-response association (Egbert and Barandiaran, 2014, see also Barandiaran and Di Paolo, 2014). In agreement with this effort, we draw upon a new conception of habits in the present paper that is derived from Dewey. Matthew Egbert and Xavier Barandarian have proposed a “holistic” view of habits as “self-maintaining patterns of behavior that share properties in common with self-maintaining biological processes, and that inhabit a complex ecological context, including the presence and influence of other habits” (Egbert and Barandiaran, 2014, p. 1). Their conception of habits is holistic insofar as it assumes that perception and action are two sides of the same process, occurring habitually rather than through mental representations. However, they do not take into due consideration the role of affectivity as a third intimate factor in action and perception. Therefore, we propose a “transactional,” “ecological” model of affective habits that radicalizes the role of the natural, social, and cultural environment in shaping habitual behaviors. By “transactional” and “ecological,” we mean a model positing the negotiation between the human organism and the natural and naturally social environment of which it is a part as a constitutive feature. The pragmatist tradition attributed a fundamental role to habits in nature. As emphasized by Frank Lorimer, habit formation is involved in the dynamic of building a provisional equilibrium within each organism's interactions with the environment (Lorimer, 1929, p. 12), giving them their mutual constitution, although to a differing extents and at different times. This broadly biological stance is the reason why in the first pages of his *Human Nature and Conduct*, Dewey compares habits and physiological functions, because they are both “functions of the surroundings as truly as of a person” (Dewey, 1983, P. 15). He draws an interesting parallel between breathing and digesting on the one hand, and walking and speech on the other, to support the claim that both the former and the latter require transactions between organic energies and environmental features. Just as breathing depends on lungs and the air, walking is a function of the length of one's legs and agility, as well as of the ground's structure. This means that bodily characters contribute to the constitution of habits as much as the surroundings' material conditions. The second example is even more challenging: Just as digesting is an affair of the stomach as much as of food, speech “demands physical air and human companionship and audience as well as vocal organs” (Dewey, 1983, p. 15). Evidently, linguistic habit shaping is the function of a variety of factors: physiological predispositions, organs, and the highly social structure of the human environment. Given Dewey's continuity thesis on natural and cultural features within the human world (see Dewey on cultural naturalism in Dewey, 1991, p. 48), habits are constituted by social and cultural features of the human environment as well as by physiological conditions.

In accordance with this view, we suggest understanding habits as “more or less flexible channelings of both organic energies and environmental resources,” including the human niche's socio-cultural features (Dreon, Human Landscapes: Contributions to a Pragmatist Anthropology, under review). This conception, we argue, is not only able to overcome the dichotomy between neurological processes and psychological or mental features, but can also incorporate the function of the human socio-cultural environment in shaping habits of action, feeling, and thought^17^. Anti-dualism and continuity between natural and socio-cultural features are two pivotal points that prove to be particularly advantageous when dealing with sensibility and adopting a situated view of affectivity without denying the brain's role. From this perspective, neural processes are clearly important features of a specific action or a peculiar emotion, but they are not in themselves the most decisive factors. Other aspects play a significant role in shaping one's habits of action and/or feeling, such as bodily characteristics, training, and capacities, as well as already existing practices within a specific social context and the related meanings associated with them. All of these elements are important factors within a specific channeling of the resources involved in each organic-environmental transaction by which the organism and the environment are dynamically and mutually (even if at different degrees) constituted. For example, many different aspects—both organic and environmental—provide an important contribution toward shaping teenagers' more or less provocative ways of behaving at home: an aggressive habit is a function of their suddenly augmented bodily strength and vocal tone and timbre, as well as of the gestures, demands, and responses from their parents.

We would argue that adopting this view of habits as the scaffolds of sensibility is advantageous in that it avoids a still dualistic reading of the situated affectivity approach as opposed to the preeminent internalist, brain-centered approach. We should no longer oppose the “inner” features of emotions to their “outer” conditions: the conditions of habits appear to be a key concept for overcoming any dualistic residues and for assuming that the discriminations between intra-organic and extra-organic factors are largely functional, which is to say context-dependent. They should not be regarded as ontologically primary and as grounding hierarchies that make certain features allegedly more decisive than others—neither by considering emotions to be determined by brain programs nor by seeing them as something culturally constructed and induced.

There are at least two other reasons for supporting a broadly Deweyan conception of habits when considering their connections with human sensibility and the emotions. On the one hand, this conception helps overcome the dualism between individual behavior and social practice through a theory of habit acquisition occurring “at a largely pre-personal level, by matching the practices, issues, and interlocutors we find in the already broadly habitualized social environment we belong to and interact with from birth” (Dreon, Human Landscapes: Contributions to a Pragmatist Anthropology, under review). Evidently, there are cases where a habit is acquired by the repetition of an initially voluntary act—as when, for example, students learn to type on their laptops to do their homework or to buy something on the Internet. However, most habits, particularly in early infancy, are acquired by affective attunement to the social context. Consider the case of cry modulations in infants. We suggest considering them as affective habits because they convey bodily feelings that are fashioned through natural and social conditions, i.e., they are more or less flexible ways of channeling both organic and environmental resources within specific situations and transactions. Infants do not only cry differently because of different needs—for example being hungry or angry, wanting warmth and protection, being annoyed by a stomachache, or the lack of a caregiver. Very early on, infants can attune their crying to their interlocutor; they cry differently depending on whether they are with their mother or father, and when they are in the presence of a third person, depending on whether this individual is a stranger or someone bringing them a bottle of milk. As emphasized by Mead, imitation cannot be assumed to be a key concept in the acquisition of habits of conduct and feeling (Mead, 2011). Rather it is better to adopt an ecological approach by considering the human environment to be naturally social at one's birth and already characterized by ways of doing things and of being affected by situations, things, and other persons that pre-exist each individual's act. In other words, the human environment is already strongly habitualized, and habit acquisition largely occurs in an individual via attunement, accommodation, and responses to environmental invitations, affordances, or even dissuasions to feel, think, and act that are already enacted by the family or community group to which a young person belongs.

In fact—and this leads us to a further reason for supporting a broadly Deweyan conception of habits—the role of the agent in habitual behavior comes to the fore in the changing of habits, such as when an old habit of action, thinking, or feeling becomes obsolete and enters into crisis because it is no longer capable of responding to a new situation. This means that widespread feelings can change and be modified, whether for better or worse. It also means that habits are not impermeable to cognition, but are (or can be) a way to intelligently re-orient one's own action. For example, racist epithets and hate and diffidence toward immigrants are becoming increasingly frequent in Europe. However, it is not unusual to meet people from the Lega Party in Northern Italy who have developed an almost protective attitude toward migrants because they have come to know their personal story or have worked with them. Arguably, they changed their specific habits of thinking, feeling, and interacting with regard to immigrants^18^. In a nutshell, Pragmatism provides a theory of habit change that is crucial when assuming that habits are pervasive in human conduct and cannot be put aside by purely rational decision-making. As pointed out by Dewey, habits not only become routine when they grow to be fixed and dull, tending to replicate the same behavior even though the conditions at work have changed. Habits can also be intelligent (Dewey, 1983) when they are flexible and capable of responding to a different situation, i.e., when they maintain the peculiarly high degree of plasticity and relative indeterminateness that is typical of human transactions with the world.

Based on these premises, we will explore the (inter)connections between habits, sensibility, and the emotions in two different ways in the following sections. First, we will deal with the general thesis that sensibility is scaffolded by habits, and we will fine-tune the features of our account of affective habits. Second, we will recover Dewey's (and Darwin's) claim that an emotion can arise when there is a crisis in habitual action. We believe that this is an important insight because it sheds light on the connection between emotion and cognition, as well as on the possibility of changing one's own habits of feeling, a point clearly involving broad political consequences.

## Affective Habits

In this section, we will consider the general thesis that sensibility is scaffolded by habits, which is to say by ways of feeling, acting, selecting, knowing, and believing. Affective habits are all kinds of habits that (a) play an essential function in prompting human sensibility. They are then in turn (b) produced, nourished, and reset by our affectively charged transactions with the world. We therefore call “affective habits” those habits that scaffold sensibility, and which at the same time are supported by our sensibility. By speaking of “affective habits,” we do not wish to introduce a new kind of entity in the taxonomy of affective states. Rather, we are presenting “affective habits” as a hermeneutical tool that helps analyze affective experiences in both their passive and active dimensions. In particular, this tool allows us to see that sensibility is not just scaffolded by habits, but that it also scaffolds them. Let us recap what we said in section Affectivity, Sensibility, and the Emotions about sensibility and in section A Deweyan Conception of Habits about a pragmatist conception of habit, so as to shed further light on our motivations for employing this notion, instead of simply speaking of affective responses, for example.

Sensibility involves ways of acting that partly stabilize bodily reactions and are partly acquired from one's own group unreflectively, yet refashioned through one's own life history. But this does not mean that sensibility is a matter of discrete reactions to discrete stimuli. Rather, human sensibility is largely habitual, including as it does relatively standardized ways of selecting stimuli within the environment, as well as ways of reacting that are both organic and socio-culturally established. From this point of view, emotions often appear to be breaks in the continuum of habitual ways of feeling and acting when significant changes in the surrounding context occur. Complementarily, emotions and feelings contribute to shaping and consolidating habitualized behaviors and practices, deeply affecting the stabilization of norms and customary morality. Their influence on individual conduct is so powerful because they involve organic-cultural entanglements that anchor significant actions in bodily postures, sensory-motor agency and vice versa.

As we will see in a moment, the bidirectionality implied by this conceptualization of sensibility is fundamental because it allows us to grant a positive and transformative role to affective habits, and thus to respond to the criticism that habits are merely conservative and routine forces.

Affective habits are essentially social: they are set up in social settings and simultaneously enacted by the agent in her affectively charged transactions with the environment. The process of affective habit generation is thus efficiently circular because it emerges within the recursive transactions between agent and environment. This means that affective habits are neither the result of a particular conscious process of carving out one's own affective style, nor of one-sided social conditioning by the environment. They are the product of affectively charged transactions with the social world—a naturally social one, as stated by the Pragmatists.

Habits require a constitutive “cooperation of organism and the environment” (Dewey, 1988a, p. 15). For affective habits, this means that human ways of feeling are scaffolded by the environment, including common practices and feeling attitudes as well as existential conditions that are already there—be they good or bad habits. At the same time, this means that the agent's affective needs and interests are an integral part of the carving out of the environment.

For heuristic purposes, let's first focus on the process that runs from the environment to the organism. Human habits are social functions (Dewey, 1988a, p. 15): they are set up, nurtured, and revised within a social setting. They are functional for acting in this social environment. This means that affective habits are embedded in social settings and perform pragmatically oriented actions in affording these environments. Consequently, affective habits cannot be considered the simple outcome of organic factors, but must rather be regarded as forces that channel social energies.

Consider the affective habit of feeling suspicious and wary when you meet something new—let's say an Indian dish you have never tasted before if you are Italian. Your affective habit of suspiciousness derives from the culture you are born in, such as a closed society distrustful of cultural differences, as well as from the physiological conditions of your stomach; for instance, if you're suffering from gastritis and have experienced discomfort in the past after eating unusual kinds of food. But we can easily imagine a different scenario. For example, you might display an affective habit of curiosity and excitement when having the chance to taste an Indian dish. Many reasons may lay at the basis of this different habit—such as being accustomed to eating curries and tolerating spicy food from an early age, since your mum is a fan of Indian cuisine and thus you are used to eating different kinds of food. Or, it could simply be because you live in a multicultural city like London, São Paulo, or Singapore where there are diverse restaurants everywhere. Therefore, what comes into play in the affective habits of feeling suspicious or, on the contrary, feeling curious when encountering novelties are both their organic features and the culture in which you live.

From a pragmatist point of view, “culture” should be envisaged in its concrete form here, as well as in its continuity with nature, i.e., as including the natural environment insofar it is modified from the inside by human interventions. Culinary preferences and disgust (a highly debated topic in recent years: see for example Menninghaus, 2003; Korsmeyer, 2011) are clear cases where organic and socio-cultural aspects are channeled into affective habits, whereby it becomes difficult to trace a boundary between the two features. As Dewey wrote, there is the co-presence of “a society or a specific group of fellow-men” in habits (Dewey, 1988a, p. 16) because of the human species' conditions of stark dependence at birth. Therefore, what really matters is the specific location involved in the generation of affective habits, such as living with your mother who is a fan of Indian food, thus nourishing your culinary open-mindedness, as well as gradually accustoming your stomach to spicy food. Dewey said that usual ways of feeling, acting, and thinking are already present at birth and shape the child's environment^19^; for the most part, even adults are embedded in broadly affective habitual practices without focusing on them. (Slaby, 2016) uses the expression “mind invasion” to describe this power of the environment to shape and modulate individual affective styles contrary to the individual's prior orientations. He adduces the example of workplace affects, whereby interns “have to habituate in various informal ways (…) in order to become ‘one of them,' where this being ‘one of them' will crucially include numerous forms of affective comportment and a particular affective style.” (Slaby, 2016, p. 1) Pivotal work has been done on this aspect by Pierre Bourdieu, who has emphasized how far the habits of one's own social group of belonging—the class habitus—can shape individual conduct and actions by affecting even one's own preferences and refusals at a pre-reflective, bodily level (Bourdieu, 1979).

This is crucial because it clearly explains that it is not the case that an agent feels something *ex nihilo* and then repeats this feeling until it becomes an almost automatic part of her affective behavior. Rather, her actions and transactions are already embedded in a specific affective context of practices and meanings which shapes them. This involves explicitly giving up methodological individualism as a privileged approach to habits by claiming that “a belief of exclusive ownership” (Dewey, 1988a, p. 15) is misleading. De Beauvoir (2012) said that one is not born, but rather becomes, a woman. Bringing de Beauvoir's pivotal insight into our topic means that women's ways of feeling, affective gestures, and desires are shaped by the society into which they are born. It is society that equips them with the affective style proper to a woman.

But we need to be careful. Our model of affective habits, which stresses their social-normative character, is not another culture-dependent view of sensibility. Rather, we derive from Dewey the claim that habits are a channeling of both organic and environmental resources. Consequently, even if the weight of the cultural norms that condition the ways women move, feel, think, and speak within different social contexts is of huge importance, the fact that women can get pregnant (or sometimes that they cannot) strongly affects their sensibility and contributes to shaping it.

We also wish to distance ourselves from a conservative view of affective habit generation as the result of society's ubiquitous conditioning powers. As Slaby rightly pointed out, there are many cases of the exploitation of affective habits in certain contexts our affective societies, ranging from the workplace to marketing and advertising sectors^20^. As philosophers and pragmatists, we need to denounce this. But this is not a universal rule. In pragmatist terms, habits are apt to change, and they play positive roles in shaping the environment. We will discuss habit change in the next section. Here we will consider the carving out of the environment in terms of affective niche construction.

This also means that we are not required to see the process of affective habit generation as one-sided. Therefore, let's consider the other direction of the process we have already introduced at the beginning of this section, which goes from the agent (whether it be an individual or a group of people) to the environment. Arguing for the radical embeddedness of affective habits means focusing not simply on the setting, but also on the interactions with a group of people to which an individual belongs. Therefore, this radical embeddedness is a matter of having a transaction with an environment—and not just of being influenced by it. It follows that individual sensibility can shape habits by virtue of their transaction with a social setting, which is the case when we regulate our habitual affective moods, for example, by listening to rock music and drinking coffee if one wakes up in the morning and is feeling sluggish and does not want to go to work. There is already much literature on affective scaffoldings, so there is no need for us to dwell on the topic. What should be highlighted here is the fundamental role of an agent's transactions with a social environment in the generation of affective habits.

Colombetti and Krueger (2015) have described the features of this carving out of an environment as affective niche construction. Affective niche construction is the active manipulation and transformation of an environment in response to the agent's affective needs and interests. This affective coupling with a social environment is triggered by the agent's sensibility, i.e., in line with situated affectivity or—we might also say—with pragmatic sensibility. Sensibility is action-oriented and makes humans feel what they care for most. Once again, this means that agents are not just absorbing society into their affective habits, but are at the same time crafting society insofar as this is oriented by their sensibility. Agents can transform a social environment from within by being embedded in it, i.e., by transacting with it. Affective habits are therefore “ways of using and incorporating the environment in which the latter has its say as sure as the former” (Dewey, 1988a, p. 15).

To sum up, we have highlighted the fact that agent-environment transactions are always affectively charged by recalling that for Dewey, “transaction” refers to the structural forms of cooperation between an organism and its environment (see Quéré, 2016 on this point). These transactions underlie the generation of affective habits, insofar as they are enacted within an integrated system of mutual relationships. Sensibility and habits are strictly intertwined in organic-environmental transactions, although for the sake of analytical clarity we have distinguished two movements: one from habits to sensibility, the other from sensibility to habits. As Dewey stated in his *Significance of Emotions*, “I suppose one is fairly entitled now to start from the assumption of the sensory-continuum, the ‘big-buzzing-blooming confusion,' out of which particular sensory quales are differentiated. Discrimination, not integration, is the real problem” (Dewey, 1971, p. 179).

One last point should be mentioned before moving on to habit change in the next section. This capacity of affective habits to carve out the environment should not be restricted to the “affective zone.” Affectivity can shape cognition. Notably, Maiese (2016) has argued that an affective niche also enables the realization of specific cognitive capacities. She highlights that affective habits of framing and selective attention scaffold cognition. One example she adduces is a musician who understands herself and her surroundings in new ways while regulating her moods by playing an instrument^21^_._ Self-understanding is undoubtedly a cognitive process and, in this case, has been activated by the affective habit of playing a musical instrument for mood regulation. This case is of paramount importance because, as we will see in the next section, that a revision of habits is possible precisely because of emotions. Habit revision clearly brings a cognitive dimension into play.

## Habit Crisis and Emotions

A specific field of inquiry with regard to the connections between emotion and habits is the breaking or crisis of already existing habits, which generally occurs when an agents' previously habitual ways of behaving no longer fit a given context. We will tackle the issue by recovering some insights from Dewey and then developing the consequences of this view with reference to the cognitive value of emotions and their role in habit change.

As claimed in the previous sections (A Deweyan Conception of Habits and Affective Habits), from a Pragmatist perspective, habits appear to be largely socially acquired, often at a pre-personal level through adjustments with already existing practices and an affectively grounded mutual coordination of gestures oriented toward an action goal—either a common one or one that is an object of contention. Regarding the topic of habit change, it is useful to consider Dewey's engagement with Charles Darwin's third principle for explaining emotion, with reference to so-called cases of “direct nervous discharge.” According to Dewey's interpretation, Darwin gathered “idiopathic” emotions apparently lacking a specific cause and characterized by an overflow of organic energies under this label, even though the principle of the direct action of the nervous system does not deny that autonomic changes can have a cause, such as when one blushes because of being ashamed (Darwin, 1872). In contrast, Dewey considered these emotions to be “cases of the failure of the habitual teleological machinery” (Dewey, 1971, p. 139), where a previous existing habit is no longer suited for a novel or modified situation.

Consider, for example, scholars who are presenting a talk or teaching in class using slides projected on a screen. Something may go wrong: the Wi-Fi connection may not work, perhaps they forgot their flash drive at home, or maybe a student asks them an odd, unexpected question. The situation can become more or less disruptive, depending on the lecturer's past experiences, the audience's degree of patience, and so on. In any case, a certain degree of anxiety, embarrassment, and disorientation will typically occur. The situation can be easily resolved if the lecturer is an experienced speaker, but it can also give rise to a small personal crisis: through this distress, the scholar may become aware of the extent to which her thinking is dependent on fixed formulas or rigid argumentation schema. She may then problematize her habits themselves and try to modify them so as to render her teaching more vivid and clear. This is where a form of behavior that has become customary (in current academia) can be reflected on and revised, or even replaced by a new habit of teaching.

Consider the crisis of linguistic habits between different generations and/or between diverse linguistic registers and social contexts. Teenagers typically develop a jargon by spending time with their peers, chatting on the web, or playing online video-games together. They absorb from their peers not only a particular way of speaking but also modes of interacting, responding, and engaging with their partners. These are felt as obvious within their social group and nourish their sense of inclusion and belonging—or exclusion with reference to those who do not share their habits of speaking, feeling, and acting. When speaking with an adult, they tend to push their speech toward a more controlled use of words. But sometimes they are unable to perceive that a certain mode of speaking—not only the choice of words, but also their tone of voice, a certain melodic line, interjections, and bodily gestures—can be perceived as aggressive, offensive, and confrontational by an adult. This can lead to a quarrel, a state of anger and frustration, or a feeling of having being misunderstood. This kind of affectively grounded awareness can emerge because of an unexpected answer by the adult interlocutor (a mother's angry answer or a critical note from a teacher) that tends to produce an individual reconsideration of a customary behavior and a redirection of the previous habit. Teenagers may then either drop their old mode of behavior and replace it with a gentler way of expressing themselves, or reinforce the previous habit if they feel that they are not receiving the acknowledgment they deserve from adults.

Generally, we could state that a crisis in habitual transactions with one's own environment and social group for the most part gives rise to an emotion that, on the one hand, manifests the habit crisis, and on the other elicits a revision of the habit itself. This revision consists of a more reflective attitude whereby the individual adopts a more responsible role in the literal sense of the term—that is, it occurs when and because the agent is obliged to respond to a changed situation that frustrates her previous ways of behaving.

A series of implications derive from this approach to habits and emotions as strictly intertwined aspects of behavior. First, affective habits, while being largely pre-personal and pre-reflective, are not at all foreign to cognition, but rather continuous with it. An emotion caused by a crisis in habitual behavior will either confirm old habits of thinking or elicit their revision, reorienting the person toward an alternative solution to her problem: it “opens the eyes” of the individual to what is no longer functioning as it used to and boosts the search for alternative modes of thinking. As is well-known, a reflective process of inquiry, for the Pragmatists, cannot begin in the void, from an artificial doubt (Peirce, 1868). There must be a real doubt for a cognitive process to arise and such doubt only emerges when one no longer knows what to do, when one's habitual behavior no longer works because it does not fit a changed situation. Hence, the transaction must be re-directed so as to produce a new belief or habit of action (Peirce, 1877). In a nutshell, the breaking of an old habit and the establishment of a new one lie at the core of cognition for the Pragmatists. Our point here is to emphasize that this break does not happen to a mind that is fundamentally non-affective. Consequently, it is urgent and real insofar as the life of fully embodied and embedded beings is at stake, to a weaker or stronger degree, when a habitual transaction is disrupted. Their interests, desires, and needs are at play and will constitute a crucial criterion for orienting and checking the efficacy of a specific process of thinking, as suggested by Dewey through his idea of “affective” or “qualitative thought” (Dewey, 1984, 1988a).

Second, affective habit crises can engender an affectively grounded form of awareness and self-reflectivity in which one feel's her own part and responsibility within a shared action and (either peaceful or aggressive) interaction. This is particularly clear in Dewey's conception of the moral process as a transition from “customary morality” to “reflective morality” (Dewey, 1985). Customary morality consists of pre-existent social habits and norms, widespread sensibility, common preferences, and refusals pervading the environment to which one belongs. These entrain one's behavior very early on. In this deflationary sense, it can primarily be understood with reference to reflective morality, consisting in the process of revising and rejecting the habits of action, feeling, and thought one has inherited and absorbed from one's social group, or in the adoption of new and more conscious ones because one's usual ways of behaving have entered into crisis and been disrupted for different reasons. Individual decision-making occurs against the largely habitual-affective background of pre-existent customs and consists in the capacity to re-invent one's own actions through imaginative, affective, and cognitive resources (Dreon, 2020).

To conclude, habits can change and are often disrupted in ordinary life. Of course, habits can be conservative and exert a regressive effect on social action (James, 1983). However, Dewey's work helps us realize that they are also flexible and diverse, and that they can be changed not merely through an allegedly voluntary act of the will exercised by pure consciousness, but when the conditions for their existence change—such as the organic conditions or the environmental context—because of either individual impulses or unexpected events. Both the moment of crisis and the phase of reconstruction are charged with affective value given that the transactions at stake are not merely contemplative, but have an impact—be it big or small—on the life of the organism.

## Conclusion

In this paper, we have provided a pragmatist conceptualization of affective scaffoldings as habits. We have employed the notion of affective habits to depict the main features of this conceptual shift. We will now summarize what we believe to be the main benefits of our proposal.

*First*, we have an active (channeling of energies) and transformative view (habit change and revision) of affective scaffoldings. This is an ameliorative move because it allows us to respond to an exclusively conservative view of habits. Thus, our proposal offers an open, pluralist, and transformative conceptualization of affective habits that we think can serve as a starting point for further research on their ethical and political dimensions.

*Second*, our account helps to understand the relationship between the pre-reflective dimension of affectivity (very much stressed in the literature on affective scaffoldings) and cognition in terms of continuity. By looking at the role of emotions in habit crises, we have especially highlighted the role of affectivity in framing habits of thought by making essential reference to the work carried out by Dewey in engaging with Darwin. We believe that this is beneficial for further developing the research on situated affectivity without neglecting the cognitive dimension that is crucial in the intertwining of sensibility and the environment.

*Third*, by proposing affective transactions at the core of the creation of habits, we have stressed an aspect that, although present, has not received sufficient attention in the literature on affective scaffolding, namely, the view of affective habits as ways of interacting. We think that this shift is crucial for avoiding the risk of understanding affective scaffoldings in terms of resource employment, thereby definitively eradicating dualistic residues. This last point led us to the main feature of our approach, namely the attempt to understand affective habits as something “in-between” —at once organic and cultural, individual and social, pre-reflective and cognitive, routine, and transformative.

In this paper, we have advanced a specific conceptualization of affective habits as relatively flexible ways of channeling affectivity. By suggesting that affective scaffoldings be understood as habits, we do not lose any phenomenological accuracy. On the contrary, we can get a broader and more nuanced picture of our affective transactions with natural and social environments.

We believe that a strong emphasis on the intertwining of sensibility and habits could have significant consequences for interpretations of our present society. On the one hand, considering that sensibility functions in a habitual manner means assuming that, as in one of our examples, racial prejudices are nourished by habitual actions while also reinforcing discriminatory habits. From this perspective, habitual sensibility certainly involves a regressive dimension that can explain some of the most dangerous social conditioning processes. On the other hand, however, our approach allows some room for change that is not driven by pure will but comes from the transformation of those conditions that constitute habits, with regard to both agents and society. For example, when some of the racists mentioned above become old and require assistance from strangers, since their former intimate social relations are gone, they will feel vulnerable and bond with their caretakers. This change of existential condition can trigger a change of habits and prompt a change of sensibility toward foreigners. Of course, there is no guarantee that habits will change in a pro-social way: dependence on other people can also reinforce prejudices, resentment, and hostile actions. But our point is that habit change happens—and it happens every time there is a transformation of either the agential or environmental features constituting a habit. Furthermore, we have also stressed that habit change is possible; it cannot be excluded *a priori*. And this means that we can activate processes of habit revision at both the individual and the societal level.

## Data Availability Statement

The original contributions presented in the study are included in the article/supplementary material, further inquiries can be directed to the corresponding author/s.

## Author Contributions

Sections Introduction, On Affective Scaffoldings and Their Theoretical Background, Affective Habits can be ascribed to LC and sections Affectivity, Sensibility, and the Emotions, A Deweyan Conception of Habits, Habit Crisis and Emotions to RD. The section Conclusion has been jointly drafted and thus can be ascribed to LC and RD. All authors conceived, discussed, and revised the manuscript.

## Conflict of Interest

The authors declare that the research was conducted in the absence of any commercial or financial relationships that could be construed as a potential conflict of interest. The reviewer PC declared a past collaboration with one of the authors LC to the handling editor.

## Footnotes

^1^In doing so, they have followed the path traced by the transactional accounts of emotion. See Parkinson (1996) and Parkinson et al. (2005).

^2^Although we cannot go into the rich debate on emotional content here, it is important to mention that Shargel and Prinz (2018) have recently suggested an enactivist theory of emotional content developed out of Griffith and Scarantino's view of emotions. Shargel and Prinz claim that if emotions are affordances, then it is possible to argue for a direct relationship between emotional embodiment and emotional content which gives credit to the embodied accounts of emotion. However, Shargel and Prinz have also highlighted some limits to the notion of affordance as an invariant feature of the environment to be perceived and of emotion as a representational state. If emotions as affordances are still representational states, they cannot fully capture the contribution of emotional embodiment. Shargel and Prinz have thus reframed their claim in enactive terms, which state that emotions do not *represent* affordances, but *create* affordances as new possibilities for action. Rietveld (2008, 2012) has challenged this standard interpretation of affordances as being in some sense objective. For Rietveld, affordances are not mere action possibilities, but “bodily potentiating and/or experienced as having an affective allure” (Rietveld, 2012, p. 212). This view of emotions as skilful, unreflective actions is another important line of investigation that can be drawn from Griffith and Scarantino.

^3^A fundamental reference for this proposal is the work on niche-construction carried out by Sterelny (2010).

^4^A noteworthy exception is Michelle Maiese, who looks at dance therapy and peace-building practices as affective scaffoldings (Maiese, 2016). Candiotto and Piredda (2019) too have argued that we need to primarily focus on affective practices, instead of objects. But since their paper is not devoted to affective scaffoldings in particular, their proposal requires further development in this regard.

^5^For a critical interpretation of the user-resources relationship, see Slaby (2016) and Candiotto and Piredda (2019).

^6^Another way to avoid this risk is to look at affective scaffoldings from the perspective of affordances, although not according to the standard interpretation of affordances as objective properties of the environment. The reference is to Rietveld and Kiverstein's new conceptual framework for understanding affordances on the basis of exercising an ability in a specific context (Rietveld and Kiverstein, 2014). We cannot develop the interesting overlaps between our pragmatist solution and the notion of affective scaffolding grounded in affordances. These overlaps certainly deserve attention in future works, but such an inquiry clearly goes beyond the limits of this paper because it would involve an evaluation of the extent to which a revised and extended concept of affordance (Rietveld, 2008; Rietveld and Kiverstein, 2014) and a Deweyan notion of habit converge and differ.

^7^We cannot argue for this thesis here, but we plan to publish a paper on it in the near future. See also Di Paolo et al. (2018) about that.

^8^We have found a deep convergence with what Maiese (2016) has said about affective framing habit, even though she makes no reference to the Pragmatists.

^9^In the contemporary philosophical literature, which is very much influenced by cognitive science, “emotion” is often used as the key term to define the object of inquiry. In cultural studies, by contrast, “affect” is the privileged term (see Slaby et al., 2017). Slaby (2016) has put forward an inclusive framework for understanding affective phenomena stretching from categorical emotion types to moods, background feelings, and atmospheres. Giovanna Colombetti (2014) has argued for the primacy of affectivity as the primordial way in which an organism makes sense of its environment.

^10^See Frijda (1986) and Lazarus (1991) that are among Colombetti's references in this regard.

^11^In *Art as Experience* (Dewey, 1989), Dewey makes an insightful point on a related issue when dealing with the long-standing problem of the properties of a picture. According to the traditional approach to the problem, the question is: When experiencing a picture as sad, is sadness a property of the picture or must it be ascribed to the subject, who would be projecting his or her own mental state on an exterior object? If sadness is an attribute of the picture—so the argument runs in the 20th-century debate on aesthetic qualities – can sadness be reduced to the physical properties of the picture, or is it supervenient on the character of the brush strokes and the bidimensional nature of the canvas (Sibley, 1959, 1965)? Dewey's answer is that the whole question is ill-posed: Being sad is a characteristic of the relation—a fully real one—between an organism (more specifically, a naturally cultural organism) and its own environment, embracing previous organic-environmental transactions (including cultural and meaningful transactions in the case of humans) and which is continuously transformed by new transactions.

^12^This conceptual shift from interaction to integration can be seen in cognitive science as well. The more recent integrationist models in neuroscience show that emotion and cognition are deeply entangled in our mental life (Gray et al., 2002; Lewis and Todd, 2005; Pessoa, 2008, 2013). The integrationist model has now been adopted in many specific research fields, from developmental psychology (Labouvie-Vief, 2015) to theories about learning and skillful behavior (Gardiner, 2015).

^13^Incidentally, we should note that there is a convergence between Dewey's mature preference for the word “transaction” over “interaction” and De Jaegher and Di Paolo's emphasis on structural coupling and participatory sense-making in their claim that enactivism is not interactionism (De Jaegher and Di Paolo, 2012). On the similarities and differences between pragmatism and enactivism, see Dreon (2021).

^14^In using “proto-valuation,” we make reference to Dewey's famous distinction between “valuing” and “evaluating” in his essay “Theory of Valuation” (Dewey, 1988b). Valuing consists in a form of immediate refusal or acceptance, praise, or blame, while evaluating involves a more reflective attitude. However, it is important to emphasize that in Dewey's view, the emotions play an important role in both processes. Consequently, these processes (valuing and evaluating) should be considered as parts of a continuum and not as opposing allegedly irrational valuations to cognitive evaluations. Here we are referring to the first function, namely how emotions can provide a direct valuation in terms of acceptance and refusal. In section Habit Crisis and Emotions, we highlight the evaluative function played by the emotions in situations of crisis in habitual behaviours. Accordingly, emotions work as a source of more conscious forms of facing problematic circumstances. Therefore, they can have an epistemic function.

^15^Giovanna Colombetti (2007), in this regard, has sharply remarked that we cannot split appraisal and arousal in the affective experience, as has been done by the cognitivists. This means that the evaluation enabled by the emotions is largely constituted by felt experience. Works on affective valence have in fact showed how much felt experience is already a form of evaluation (Charland, 2005; Colombetti, 2005). Although we employ the concept of “proto-valuation” as specified in footnote 12, we find an important reference to the enactive conceptualisation of “proto-evaluation” by Colombetti because it involves a clear distance from and an alternative to a cognitivist view of emotional evaluation. In this regard, see also Helm (2002, 2009) and Döring (2007), where pleasure and pain constitute a distinctive and intrinsically motivating evaluation that is shared with emotions and desires.

^16^At present, there are a good number of works on Dewey's conception of habit, cf.: Colapietro (2004), Shusterman (2008), Dreon (2010), Crossley (2013), Sullivan (2013), Quéré (2016), Dreon (2016), and Steiner (2021). An interesting attempt to use Dewey's notion of habit within an enactivist background can be found in Ilundáin-Agurruza et al. (2018).

^17^The pragmatist approach to habits we are developing here shares the same aims as situated views of affectivity, for example when it is stated that situated affectivity goes beyond “the sterile debate over nature and nurture” (Griffiths and Scarantino, 2008, p. 12). This is also case in terms of its scope, for example when affordances are employed to account for socio-cultural practices in shaping the human niche (Rietveld and Kiverstein, 2014, p. 326).

^18^It can be objected that they changed their habits only regarding the specific person they met at work, and not regarding immigrants in general. However, even if this is the case, this specific habit change regarding a specific person can then trigger an overall revision of habits, especially if it will produce a new habit of interaction with the colleague.

^19^“Each person is born an infant, and every infant is subject from the first breath he draws and the first cry he utters to the attention and demands of others. These others are not persons in general with minds in general. They are beings with habits, and beings who upon the whole esteem the habits they have” (Dewey, 1983, p. 43).

^20^It is important to stress that mind invasion works against the prior orientation of the individual. Thus, this is not a general thesis about the power of the environment over the individual, but a precise analysis of a specific phenomenon: the one in which the affective habits endorsed by an individual immersed in an affective culture are detrimental for the individual in question.

^21^The example of the musician comes from Colombetti (2016). Colombetti adduces it as a case of emotion regulation, but Maiese's point is that the habit of playing the piano for emotional regulation also entails cognitive outcomes—in this case, self-understanding.
